# Iron Deficiency in Adolescent and Young Adult German Athletes—A Retrospective Study

**DOI:** 10.3390/nu14214511

**Published:** 2022-10-27

**Authors:** Rubina Roy, Momme Kück, Lukas Radziwolek, Arno Kerling

**Affiliations:** 1 Department for Rehabilitation and Sports Medicine, Hannover Medical School, 30559 Hannover, Germany; 2 Bundeswehr Medical Service Headquarters, 56070 Koblenz, Germany

**Keywords:** iron deficiency, adolescent athletes, iron substitution, competitive sports, physical performance

## Abstract

Background: Iron deficiency is a common phenomenon in sports and may lead to impaired physical performance. The aim of the study was to determine the frequency of iron deficiency in competitive athletes and to discuss the resulting consequences. Methods: The data of 629 athletes (339 male, 290 female) who presented for their annual basic sports medicine examination were investigated. Depending on age (<14 years, 15–17 years, ≥18–30 years), four groups ((I.) normal hemoglobin (Hb) and ferritin level (≥30 ng/mL for adults and 15–18-year-olds; ≥20 ng/mL, respectively, ≥15 ng/mL for adolescents and children), (II.) prelatent iron deficiency (ID) (normal Hb, low ferritin), (III.) latent ID (additionally elevated soluble transferrin receptor or decreased transferrin saturation) and (IV.) manifest anemia) were distinguished. In addition, the iron status and exercise capacity of different types of sports were compared. Results: Overall we found an iron deficiency of 10.9% in male (mainly in adolescence) and 35.9% in female athletes (emphasized in adolescence and young adulthood). There were no significant differences in iron status in regard to the different sport types or in maximum performance for the different groups of iron deficiency. Conclusions: Adolescent and female athletes are more likely to have an iron deficiency. Therapy concepts for athletes therefore should pay attention to iron-rich diets.

## 1. Introduction

Most of the iron found in the human body (3–5 grams or 35–60 milligrams/kg of body weight) is bound to hemoglobin (>60%). In addition to myoglobin (approx. 10%), which like hemoglobin has a predominantly oxygen transport function, iron is also found in various enzymes, including the respiratory chain (approx. 2%); a small proportion is present in transferrin (approx. 0.2%), which regulates iron transport in plasma. Storage iron (ferritin and hemosiderin) includes 15–25%, whereby the iron stores are smaller in women [1,2]. Iron, therefore, is an essential component of maximum oxygen uptake and occupies a central role in performance metabolism. Whether depleted iron stores with still normal hemoglobin levels already lead to impaired performance in athletes is controversially discussed [2,3]. The daily loss, e.g., via intestinal epithelia or skin cells, is about 1 mg; women additionally lose up to twice the amount of men, depending on the intensity of menstruation [2,4]. In athletes, additional iron losses via the gastrointestinal tract may occur as a result of exercise-induced microbleeding in hemorrhagic gastritis/colitis as a result of microischemia during excessive training [5], often favored by the simultaneous use of nonsteroidal anti-inflammatory drugs [6]. These losses are not insignificant and can be as high as 2 mg/day after high-intensity exercise. Increased losses via sweat and urine also increase the requirement for the athlete [7].

Ferritin represents the body’s storage of iron stores; however, as an acute phase protein, it is also affected by inflammation, infections, and malignancies and therefore cannot be used without restriction in the diagnosis of iron deficiency [8,9]. In contrast, soluble transferrin receptor (sTFR) reflects the bone marrow’s demand for iron and therefore, increases iron deficiency, in contrast to ferritin, its fluctuations over the course of the day are smaller and exercise-induced changes are also more reliably indicated by sTFR [8,10].

Prelatent iron deficiency involves decreased ferritin levels without alterations in other parameters such as hemoglobin or sTFR. The physiological ferritin concentration in the untrained is approximately 20–300 ng/mL [2]. However, there is also evidence that, in athletes, the hemoglobin mass itself performs an iron storage function (in addition to the reticuloendothelial system of the liver) and the threshold values for ferritin are naturally lower in endurance athletes as a result [11].

In the latent iron deficiency stage, normal red blood cell parameters are accompanied by an increase in sTFR in addition to a decrease in ferritin levels and transferrin saturation. The sTFR is increasingly expressed when, for example, there is less iron in the bone marrow as a result of iron deficiency which then leads to increased iron absorption into the tissue. Unlike ferritin, it is not affected by inflammation or endurance exercise [12]. However, elevated levels have been described after weight training in men [13]. Increased sTFR values also occur after stays at altitude due to increased erythropoiesis caused by erythropoietin [14]. If hemoglobin, MCV, and MCH are also reduced, this is referred to as iron deficiency anemia. The cut-off value at which one speaks of a reduced ferritin value is fundamentally problematic. For competitive sports, Clenin et al. have made a reasonable classification that takes into account age and gender [15].

According to WHO estimates, approximately 2 billion people suffer from iron deficiency, a quarter of them from iron deficient anemia; the highest prevalence is found in South Asia and Central and West Africa [16]. In contrast, in Western countries, a prevalence of up to about 20% in adolescent and menstruating women and 7% in young men has been described, with a much lower proportion of manifest iron deficiency anemia of just under 2% in women and less than one percent in men [17,18]. The prevalence of iron deficiency in competitive athletes is partly described as being much higher, ranging from 15 to over 57% in women and 3–31% in men [19,20]. Favoring factors for iron deficiency are, in addition to the female gender, insufficient and low-quality food intake, vegetarian and vegan diets, participation in endurance sports, and in sports that are associated with an increased risk of eating disorders [15,21].

The purpose of this study is to analyze the iron status of competitive athletes at different ages of adolescence and young adulthood and to discuss the resulting consequences. In addition, we investigated the iron status in regard to the different sport types and the maximum performance for the different groups of iron deficiency.

## 2. Material and Methods

### 2.1. Study Design and Participants

For this retrospective analysis, the pseudonymized data of 629 competitive athletes (339 male/290 female) who presented at the Lower Saxony Olympic Training Center from May 2021–April 2022 as part of their annual basic sports medicine examination were investigated. The classification of the groups was based on Clenin G et al. [15], whereby, in addition to a group with a normal hemoglobin value and normal ferritin level, a group with manifest anemia and, in the nonanemic athletes with a low ferritin level, 2 further groups with prelatent (age-related low ferritin) or latent iron deficiency (additionally increased sTFR > 4.13 mg/L or transferrin saturation < 16%) were distinguished. The normal level of ferritin is age-dependent and accordingly, the classification of normal values was set at ≥30 ng/mL for adults and 15–18-year-olds and ≥20 ng/mL, respectively, ≥15 ng/mL for adolescents and children.

Types of sports were classified according to Pellicia et al. into skill, power, mixed, and endurance disciplines depending on cardiovascular load and competition-specific muscular contraction behavior [22]. Four hundred thirty-one athletes performed an exercise test using bicycle ergometry.

### 2.2. Blood Collection and Preparation

All blood samples (2.6 mL ethylenediaminetetraacetate (EDTA) monovette for blood count, lithium heparin monovette (5.5 mL) for serum enzymes and iron metabolism parameters) were collected in the morning via a butterfly needle inserted into an antecubital vein with the athletes lying or seated. All athletes were advised not to do exhausting training on the examination day and the day before. Analysis was performed using the routine laboratory chemical measurement procedures in the laboratory of the Klinikum Region, Hannover GmbH.

For this study, the blood count and the parameters of iron metabolism were taken into account. Because of influence, ferritin levels athletes with an increased c-reactive protein were excluded. The soluble transferrin receptor and the transferrin saturation were additionally determined only at a ferritin value < 35 ng/mL.

Hemogram was processed with an XN1000 or XN 2000 Analysis system (Sysmex GmbH, Norderstedt, Germany) where hemoglobin was measured by sodium lauryl sulfate (SLS) hemoglobin method; hematocrit, erythrocytes, leukocytes, and platelets were analyzed by hydrodynamic focusing and flow cytometry with semiconductor laser. MCH, MCV, and MCHC were calculated as red blood cell erythrocyte indices. For iron status measurements, the lithium heparin monovette was centrifuged at 3000 rpm for 10 min (Rotanta 460 Robotic, Hettich GmbH & Co., KG, Tuttlingen, Germany). Iron, transferrin, and soluble transferrin receptor were analyzed with the Cobas 502-, and ferritin with the Cobas e601 analyzer (Roche Diagnostics International AG, Rotkreuz, Switzerland). Transferrin saturation was calculated.

The evaluation was carried out in accordance with the Declaration of Helsinki and current guidelines of good clinical practice.

### 2.3. Anthropometric Data

Body height and weight were assessed in a standardized way. Body fat percentage was determined by segmental multi-frequency bioimpedance analysis (InBody720; Biospace, Korea).

### 2.4. Ergometry

To examine exercise capacity (measured as peak power output in W/kg body weight), athletes did incremental exercise on a speed-independent bicycle ergometer (Ergometrics 900 s, Ergoline, Bitz, Germany) with at least 60 revolutions per minute, under electrocardiogram (ECG)-monitoring. The tests started weight-dependent with a workload of 20 W for athletes weighing less than 30 kg, with 50 W for athletes between 30 and 50 kg and they were increased in 10 W increments each minute until the onset of physical exhaustion (peripheral muscle fatigue and/or dyspnea). Athletes with a body weight of 50 kg or more started with 50 W with 16.67 W increments per minute. Blood pressure and blood lactate concentration were acquired at rest, 1 min after the start of testing and every 3 min during the test. Capillary blood samples of 20 µL were taken from the arterialized earlobe, deproteinized, and then measured with a lactate analyzer (Ebio 6666, Eppendorf, Berlin, Germany). Only the maximum values were taken into account for the evaluation.

### 2.5. Statistical Analysis

All data are given as mean ± standard deviation. Normal distribution was tested using the Kolmogorov–Smirnov test. The distribution of the data was tested with a Chi-squared test. Differences between groups were tested with an ANOVA. All post hoc tests were corrected after Bonferroni. The significance level was set at 0.05. All calculations were done with SPSS (Version 27, Armonk, NY, USA).

## 3. Results

In our collective, iron deficiency is found in about 25% of competitive athletes, with a proportion being three times more common in women (Table 1). Manifest anemia was found in only 1.3%.

In the comparison of the 4 groups (anemia/normal ferritin levels/prelalent as well as latent iron deficiency) no significant differences between the sport types were found (Table 2).

**Figure 1:**
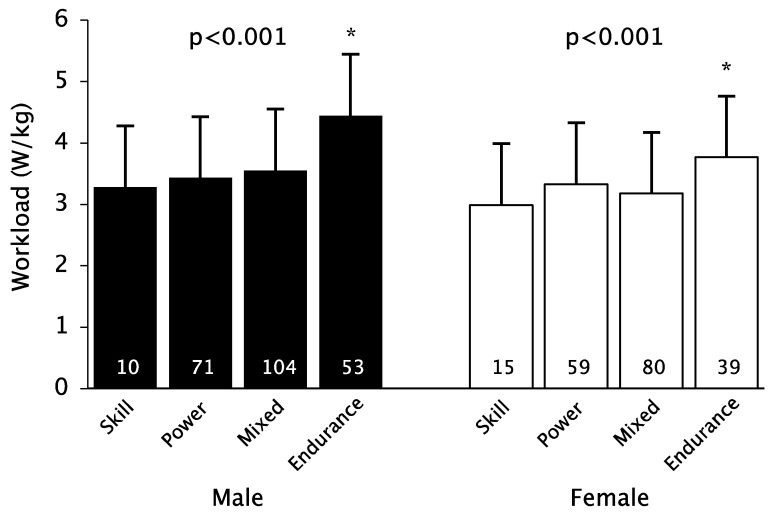
Sport types comparison of workload. * *p* < 0.05 post hoc tests Endurance vs. all other types.

When comparing the groups according to sport, the highest performance was found in the endurance athletes (Figure 1). However, there was no significant difference in workload with regard to the ferritin levels (Table 3 and Table 4).

## 4. Discussion

The main finding of our study is that about a quarter of the athletes suffer from iron deficiency, mainly women, consequently, the proportion of anemic athletes is fortunately much lower. In adult men, just one person suffered from iron deficiency. This observation is consistent with those of the studies mentioned in the introduction [1,2,4,17,18,19,20] and suggests the recommendation for regular checks of iron levels in adolescent competitive athletes; adolescent women in particular should be monitored and treated more intensively. On the other hand, we found no significant difference in performance for the different groups of iron deficiency.

Regular endurance training leads to an increase in erythropoiesis as well as an increase in plasma volume, which is relatively larger than the erythrocyte mass. This leads to a decrease in the concentration of hemoglobin, as well as hematocrit at normal total hemoglobin mass. Ferritin levels may also be lower due to dilution; MCV and MCH are in the normal range [7]. This phenomenon, known as runner’s anemia or dilutional pseudo-anemia, is mainly found in endurance athletes from a training period of 10 h/week and over ultralong endurance distances [15] and must not be confused with manifest iron deficiency anemia. It is found as a physiological adaptation response with an optimal balance between decreased blood viscosity and higher oxygen transport [23]. A distinction between prelatent iron deficiency can only be made with the rather complex determination of the total hemoglobin mass. The endurance athletes in our collective, however, were not affected more by prelatent or latent iron deficiency and anemia than the other groups.

From a physiological point of view, an effect on performance should become apparent earliest in the case of an incipient iron deficiency in the tissue, i.e., in the state of a latent deficiency, when enzymes of the respiratory chain are possibly impaired even before the occurrence of anemia. In order to avoid a potential reduction in performance, therapeutic intervention is recommended as soon as a prelatent iron deficiency occurs, whereby the first therapeutic option is an optimal supply of iron through food [7,15]. However, we could not find any difference in terms of performance between athletes with normal and decreased ferritin levels in our collective. In addition to the small group size, this could also be due to the test procedure not being specific to the types of sport. Furthermore, a possible effect of iron substitution on individual athletes over time was not investigated.

The daily iron requirement depends on various factors and is increased during growth, menstruation, and under vegetarian and vegan diets. Thus, higher intake levels are recommended for vegetarians due to the poorer bioavailability of plant-based iron (14 mg instead of 8 mg/day in men, 33 mg instead of 18 mg/day in women) [24]. In addition to iron deficiency, there is also an increased risk of deficiency of Vit.B2, zinc, and omega-3 fatty acids, in strictly vegan diets, additionally, there is a lack of fats, proteins, calcium, and iodine [25]. In adolescence, the increased demand is mainly due to the expansion of the total blood volume, the increase in lean body mass, and the onset of menses in young females. The mean total iron requirement can double to up to 2.2 mg/day or more in female athletes because of heavy menstrual periods compared to preadolescence [26]. Another problem is the female athlete’s triad consisting of low energy intake, menstrual dysfunction, and low bone mineral density, which is often found in endurance athletes and often leads to iron deficiency [27]. In this case, intensive care of the athletes is necessary, and a pure iron substitution would fall short in this case.

The daily iron requirement even for athletes with a high energy turnover can normally be covered with a normal diet. Heme iron is mainly found in animal products of mammals, poultry, and seafood and has a significantly better absorption rate (up to 35%) than non-heme iron (2–20%) [28], which is mainly found in plant products. Intestinal iron absorption can be increased depending on its blood concentration [29]. Good sources of animal iron include clams and liver; plant-based iron is highly concentrated in cooked soybeans, lentils, and spinach [23]. Athletes who supply themselves with diets other than omnivorous diets may require care from nutritionists or nutritionist counselors to ensure an adequate supply of iron.

If an improved diet does not lead to an increase in ferritin or if there is a manifest (latent) iron deficiency or anemia, oral or intravenous therapy should be considered (depending on age, type of sport, and performance status). Of the existing supplements, ferrous iron should be preferred, as it is better absorbed; a higher dosage only 3 days a week (100–200 mg as a single dose) seems to be at least as good in terms of absorption, and superior to daily substitution in terms of tolerability (fewer upper abdominal complaints, diarrhea) [23,30]. It should be taken in the morning (the slightest activity of hepcidin, which inhibits iron absorption from the small intestine) [31] and last for 3 months under ferritin monitoring. Iron supplements should not be taken together with coffee, black tea, eggs, milk, or dairy products. An interval of at least 2 h should be maintained to ensure optimal iron absorption [32]. Iron uptake can be further improved by the simultaneous administration of vitamin C (e.g., orange juice) [15].

In case of existing iron intolerance or lack of response to iron therapy, parenteral therapy should be considered. The advantages are a faster increase in hemoglobin concentration, as well as a shorter duration of treatment. Tolerability and safety have been significantly improved by new preparations, the form of application (e.g., ferinject^®^ 50 mg in 10- or 20-mL doses) also takes into account the anti-doping regulations of the World Anti-Doping Agency (WADA) [33,34,35].

It is generally agreed that anemia leads to impaired performance in competitive sports. Whether the performance of athletes with depleted iron stores but still normal hemoglobin levels is impaired is still controversially discussed; there are studies with no effects of iron supplementation [36,37] as well as studies showing an improvement in strength and endurance capacity by iron supplementation [3,38,39].

Furthermore, there is still no uniform assessment of the limit value for ferritin above which iron deficiency is present. Rubeor et al. found no improvement in performance in nonanemic athletes from iron substitution at ferritin levels above 20 ng/mL, with a possible benefit with values below [40]. This review not only studies VO_2_max but also other quantitative measurements like shuttle run time, 3000 m race pace, anaerobic speed test, etc., are considered. The already mentioned classification of Clenin et al. [15] is also very helpful in the further care of competitive athletes, as they additionally take into account age and gender for setting the cut-off values. Determination of sTFR is another method to further specify the degree of iron deficiency. To save costs (approx. 8 euros per Test), we determine sTFR only from ferritin concentrations < 35 ng/mL.

There is agreement that before altitude training to achieve the best possible effects, iron status must be optimal, but at what ferritin level this is the case is also discussed controversial (optimal ferritin level is given between 50 and >130 ng/mL [15,23]).

Regardless, the indiscriminate administration of a food supplement or drug, in this case, iron, should be rejected in the context of anti-doping education especially for young athletes so it should be noted that a routine iron substitution should not be performed. The permanent administration of iron can disguise both, the presence of an underlying disease with chronic bleeding (e.g., in the gastrointestinal tract or in gynecological diseases) and, in rare cases, even trigger hemochromatosis with appropriate genetic predisposition [9]. Therefore, drug iron therapy should always be carried out only in justified cases and should always be monitored carefully.

## 5. Limitations

Apart from the usual disadvantages of a retrospective analysis and the limitations already mentioned in the text, the following limitations can be named.

For the evaluation, it was not recorded to what extent iron substitution was already being carried out; likewise, no information that could be taken into account was available on the type of diet and the average daily iron intake on the basis of a nutrition report. A part of the study period fell in the peak phase of the COVID-19 pandemic so a performance test could not be carried out on all athletes due to hygienic measures. Additionally, the division into 32 subgroups with partly, only single, or no athletes might be too small to show significant differences. Regardless of the type of sport, all exercise tests were performed on a bicycle ergometer, and it cannot be ruled out that sport-specific testing would have revealed differences in performance between the iron-deficient groups. Furthermore, the study was performed in a German population with mainly Caucasian athletes and therefore may not have sufficient generalizability to non-western populations or non-Caucasian ethnicities. The subdivision of the sports types just illustrates the load in competition and does not reflect the cardiovascular and muscular load in the various training sessions. An additional classification of the different sports according to training hours per week has not been conducted.

The following recommendations can be made for competitive athletes

1. Athletes should eat an iron-rich diet taking into account vegetarian and vegan diets.
2. Athletes should be monitored for iron levels at least once a year, and adolescent competitive athletes and women, in particular, should be monitored and treated more intensively.
3. In case of insufficient dietary iron intake and iron deficiency, drug therapy should be taken into account (for points 2 and 3 age, type of sport, and performance status must be considered; for example, the indication of iron supplementation in a female middle-distance runner prior to international competition would be much more generous).
4. An uncontrolled iron substitution should not take place.

## 6. Future Research

Important questions for future studies are how athletes feed themselves, what percentage of the iron supply in existing iron deficiency can be adjusted by diet alone, and what percentage of athletes are already substituting iron. Furthermore, ferritin should not be used as the sole parameter for describing iron deficiency; sTFR is a good parameter to specify the degree of iron deficiency. The exercise capacity of athletes should not be determined by VO_2_max or Watt_max_ alone but in combination with sport-specific tests.

## Figures and Tables

**Table 1:** Age-group comparison of iron status.

Age		*n*	Normal Ferritin Levels	Prelalent	Latent	Anemia
up to 14	male	33	27 (81.8%)	4 (12.1%)	2 (6.1%)	0
	female	37	28 (75.7%)	3 (8.1%)	5 (13.5%)	1 (2.7%)
14–18	male	198	169 (85.4%)	14 (7.1%)	14 (7.1%)	1 (0.5%)
	female	159	97 (61.0%)	30 (18.9%)	31 (19.5%)	1 (0.6%)
over 18	male	108	106 (98.1%)	0	1 (0.9%)	1 (0.9%)
	female	94	61 (64.9%)	18 (19.1%)	11 (11.7%)	4 (4.3%)
overall	male	339	302 (89.1%)	18 (5.3%)	17 (5.0%)	2 (0.6%)
	female	290	186 (64.1%)	51 (17.6%)	47 (16.2%)	6 (2.1%)

**Table 2:** Sport type comparison of iron status.

Sport Types		*n*	Normal Ferritin Levels	Prelalent	Latent	Anemia
Skill	male	13	12 (92.3%)	1 (7.7%)	0	0
	female	17	10 (58.8%)	1 (5.9%)	5 (29.4%)	1 (5.9%)
Power	male	108	93 (86.1%)	6 (5.6%)	9 (8.3%)	0
	female	106	75 (70.8%)	14 (13.2%)	16 (15.1%)	1 (0.9%)
Mixed	male	144	128 (88.9%)	8 (5.6%)	6 (4.2%)	2 (1.4%)
	female	111	69 (62.2%)	26 (23.4%)	13 (11.7%)	3 (2.7%)
Endurance	male	74	69 (93.2%)	3 (4.1%)	2 (2.7%)	0
	female	56	32 (57.1%)	10 (17.9%)	13 (23.2%)	1 (1.8%)

**Table 3:** Subject characteristics of adolescents (age < 18 years).

		Normal Ferritin Levels		Iron Deficiency			
			*n*		*n*	*p*	g
Age (years)	male	15.6 ± 1.4	196	15.1 ± 1.3	35	0.028	0.41
	female	15.5 ± 1.6	125	15.6 ± 1.4	71	0.620	-
Height (cm)	male	178.6 ± 10.8	166	174.0 ± 11.0	33	0.038	0.43
	female	168.8 ± 7.0	99	169.9 ± 8.8	65	0.382	-
Weight (kg)	male	67.7 ± 13.6	166	63.6 ± 13.8	33	0.044	0.37
	female	61.1 ± 10.2	99	59.9 ± 9.2	65	0.420	-
BMI-SDS	male	−0.05 ± 0.77	166	−0.14 ± 0.78	33	0.564	-
	female	0.01± 0.87	99	−0.25 ± 0.77	65	0.047	0.32
Body fat (%)	male	9.6 ± 5.3	160	9.2 ± 4.7	30	0.769	-
	female	18.7 ± 7.0	99	17.9 ± 5.2	64	0.860	-
Workload (W/kg)	male	3.8 ± 0.8	138	3.7 ± 0.6	27	0.741	-
	female	3.3 ± 0.5	82	3.3 ± 0.7	56	0.588	-
Heart rate (bpm)	male	191.3 ± 11.0	138	193.2 ± 8.5	27	0.646	-
	female	189.4 ± 9.5	82	188.7 ± 8.0	56	0.643	-
RRsys (mmHg)	male	197.1 ± 25.0	138	191.8 ± 25.4	27	0.321	-
	female	183.5 ± 20.6	82	190.1 ± 22.0	56	0.073	-
RRdia (mmHg)	male	79.3 ± 9.8	138	80.8 ± 8.4	27	0.467	-
	female	79.1 ± 9.1	82	79.3 ± 8.3	56	0.927	-
Lactate (mmol/L)	male	8.90 ± 2.67	12	9.97	1	-	-
	female	9.14 ± 2.04	7	7.14 ± 1.28	2	0.143	-
Hämoglobin	male	14.88 ± 0.94	196	14.31 ± 0.91	35	0.001	0.61
	female	13.39 ± 0.85	125	12.97 ± 0.95	71	0.002	0.48
Hämatokrit	male	43.2 ± 2.7	196	42.0 ± 2.9	35	0.021	0.42
	female	39.5 ± 2.5	125	38.8 ± 2.5	71	0.044	0.30
MCH	male	29.4 ± 1.3	196	28.3 ± 1.1	35	<0.001	0.85
	female	29.3 ± 1.4	125	28.5 ± 1.7	71	0.002	0.49
MCHC	male	34.5 ± 0.9	196	34.2 ± 1.0	35	0.078	-
	female	34.0 ± 0.9	125	33.5 ± 1.0	71	0.002	0.52
MCV	male	84.9 ± 3.3	196	83.0 ± 2.6	35	0.003	0.59
	female	86.3 ± 3.1	125	84.9 ± 3.6	71	0.011	0.40
Ferritin	male	64.9 ± 32.2	196	21.1 ± 6.3	35	<0.001	1.47
	female	56.6 ±46.3	125	17.8 ± 6.6	71	<0.001	1.04
Eisen	male	106.4 ± 40.1	196	83.2 ± 29.5	35	0.002	0.57
	female	101.2 ± 38.8	125	80.8 ± 50.1	71	<0.001	0.47
Transferrin	male	274.7 ± 32.9	196	302.0 ± 32.9	35	<0.001	−0.83
	female	281.3 ± 35.5	125	320.0 ± 46.5	71	<0.001	−0.97
Lösl. Rezeptor	male	3.40 ± 0.43	18	3.66 ± 0.82	35	0.127	-
	female	3.14 ± 0.89	32	3.52 ± 1.15	71	0.028	−0.35
Trans.Sättigung	male	28.1 ± 12.2	196	19.8 ± 7.4	35	<0.001	0.71
	female	25.8 ± 9.9	125	18.1 ± 10.1	71	<0.001	0.77

**Table 4:** Subject characteristics of adults (age ≥ 18 years).

		Normal Ferritin Levels		Iron Deficiency			
			*n*		*n*	*p*	g
Age	male	21.7 ± 3.0	106	18.7 ± 0.1	2	-	
	female	21.7 ± 3.4	61	21.6 ± 3.5	33	0.632	-
Height	male	182.7 ± 9.4	81	164.0	1	-	
	female	174.8 ± 9.6	42	170.0 ± 12.5	25	0.068	-
Weight	male	78.6 ± 14.7	81	53.4	1	-	
	female	66.8 ± 11.0	42	64.2 ± 11.7	25	0.364	-
BMI	male	23.4 ± 3.0	81	19.9	1	-	
	female	21.8 ± 2.7	42	22.2 ± 2.8	25	0.555	-
Body fat (%)	male	10.8 ± 5.0	69	22.5	1	-	
	female	16.7 ± 5.2	42	18.0 ± 4.4	22	0.331	-
Workload (W/kg)	male	3.5 ± 0.8	72	3.7	1	-	
	female	3.5± 0.5	34	3.2 ± 0.8	21	0.101	-
Heart rate (bpm)	male	182.8 ± 13.4	72	181.0	1	-	-
	female	183.4 ± 11.1	34	183.6 ± 10.9	21	0.943	-
RRsys (mmHg)	male	203.3 ± 22.4	72	206	1	-	-
	female	199.1 ± 20.9	34	189.0 ± 30.0	21	0.135	-
RRdia (mmHg)	male	81.7 ± 9.1	72	91.0	1	-	-
	female	83.7 ± 7.4	34	82.0 ± 8.9	21	0.470	-
Lactate (mmol/L)	male	8.99 ± 2.90	37	-		-	-
	female	9.50 ± 2.40	17	9.28 ± 2.25	8	0.835	-
Hämoglobin	male	15.25 ± 0.96	106	12.80 ± 0.42	2	-	-
	female	13.72 ± 0.90	61	12.88 ± 0.88	33	<0.001	0.93
Hämatokrit	male	44.4 ± 2.5	106	38.1 ± 0.2	2	-	-
	female	40.2 ± 2.4	61	38.9 ± 2.6	33	0.016	0.52
MCH	male	29.6 ± 1.3	106	30.5 ± 0.7	2	-	-
	female	30.0 ± 1.3	61	28.3 ± 2.1	33	<0.001	1.05
MCHC	male	34.3 ± 0.9	106	33.5 ± 0.7	2	-	-
	female	34.1 ± 0.9	61	33.2 ± 0.9	33	<0.001	1.04
MCV	male	86.3 ± 3.0	106	89.5 ± 0.7	2	-	-
	female	88.1 ± 3.2	61	85.3 ± 4.4	33	0.005	0.76
Ferritin	male	115.8 ± 51.1	106	29.1 ± 16.6	2	-	-
	female	84.5 ± 68.0	61	19.0 ± 8.9	33	<0.001	1.18
Eisen	male	110.2 ± 41.9	106	86.5 ± 50.2	2	-	-
	female	117.3 ± 49.0	61	97.6 ± 55.2	33	0.078	-
Transferrin	male	259.4 ± 32.2	106	257.0 ± 33.9	2	-	-
	female	269.8 ± 40.5	61	328.3 ± 64.9	33	<0.001	−1.15
Lösl. Rezeptor	male	-		3.30	1	-	-
	female	2.20 ± 0.36	7	3.24 ± 1.19	32	0.006	−0.93
Trans.Sättigung	male	30.2 ± 11.2	106	25.0 ± 17.1	2	-	-
	female	31.6 ± 14.3	61	21.7 ± 12.4	33	0.001	0.72

## Data Availability

The datasets used and/or analyzed during the current study are available from the corresponding author on reasonable request.
